# The effects of real-time waveform analysis software on patient ventilator synchronization during pressure support ventilation: a randomized crossover physiological study

**DOI:** 10.1186/s12890-024-03039-0

**Published:** 2024-05-01

**Authors:** Barnpot Nakornnoi, Jamsak Tscheikuna, Nuttapol Rittayamai

**Affiliations:** Division of Respiratory Diseases and Tuberculosis, Department of Medicine, Faculty of Medicine Siriraj Hospital, Mahidol University, Bangkok, Thailand

**Keywords:** Asynchrony, Esophageal pressure, Pressure support ventilation, Patient-ventilator interaction

## Abstract

**Background:**

Patient-ventilator asynchrony commonly occurs during pressure support ventilation (PSV). IntelliSync + software (Hamilton Medical AG, Bonaduz, Switzerland) is a new ventilation technology that continuously analyzes ventilator waveforms to detect the beginning and end of patient inspiration in real time. This study aimed to evaluate the physiological effect of IntelliSync + software on inspiratory trigger delay time, delta airway (P_aw_) and esophageal (P_es_) pressure drop during the trigger phase, airway occlusion pressure at 0.1 s (P_0.1_), and hemodynamic variables.

**Methods:**

A randomized crossover physiologic study was conducted in 14 mechanically ventilated patients under PSV. Patients were randomly assigned to receive conventional flow trigger and cycling, inspiratory trigger synchronization (I-sync), cycle synchronization (C-sync), and inspiratory trigger and cycle synchronization (I/C-sync) for 15 min at each step. Other ventilator settings were kept constant. P_aw_, P_es_, airflow, P_0.1_, respiratory rate, SpO_2_, and hemodynamic variables were recorded. The primary outcome was inspiratory trigger and cycle delay time between each intervention. Secondary outcomes were delta P_aw_ and P_es_ drop during the trigger phase, P_0.1_, SpO_2_, and hemodynamic variables.

**Results:**

The time to initiate the trigger was significantly shorter with I-sync compared to baseline (208.9±91.7 vs. 301.4±131.7 msec; *P* = 0.002) and I/C-sync compared to baseline (222.8±94.0 vs. 301.4±131.7 msec; *P* = 0.005). The I/C-sync group had significantly lower delta P_aw_ and P_es_ drop during the trigger phase compared to C-sync group (-0.7±0.4 vs. -1.2±0.8 cmH_2_O; *P* = 0.028 and − 1.8±2.2 vs. -2.8±3.2 cmH_2_O; *P* = 0.011, respectively). No statistically significant differences were found in cycle delay time, P_0.1_ and other physiological variables between the groups.

**Conclusions:**

IntelliSync + software reduced inspiratory trigger delay time compared to the conventional flow trigger system during PSV mode. However, no significant improvements in cycle delay time and other physiological variables were observed with IntelliSync + software.

**Trial registration:**

This study was registered in the Thai Clinical Trial Registry (TCTR20200528003; date of registration 28/05/2020).

**Supplementary Information:**

The online version contains supplementary material available at 10.1186/s12890-024-03039-0.

## Background

Mechanical ventilation is an important life supporting treatment in patients with acute respiratory failure. After recovery from acute respiratory failure, pressure support ventilation (PSV) is the mode most commonly used during the weaning period [1–3]. PSV is patient-triggered, pressure-limited, and flow-cycled. During PSV, the ventilator is typically initiated by a traditional flow or pressure trigger system resulting from the inspiratory effort of the patient [4]. The ventilator then delivers the pressurization and is stopped when a predetermined flow cycle criterion is reached [5].

Patient ventilator asynchrony is defined as a mismatch in breathing delivery time between the mechanical ventilator and the patient [6]. Previous studies reported that patient ventilator asynchrony was significantly associated with poor clinical outcomes, including a longer duration of mechanical ventilation, a longer intensive care unit (ICU) and hospital length of stay, and increased mortality [7–10]. It commonly occurs during assisted ventilation, especially in PSV mode. Trigger and cycle asynchronies such as ineffective trigger, double trigger, premature cycling, and delay cycling, are commonly recognized in daily clinical practice [6, 11].

Identifying patient ventilator asynchrony using airway pressure (P_aw_) or airflow waveform is routinely used at the bedside; however, a previous study demonstrated that the sensitivity of these abnormal waveforms to recognize asynchrony were low [12]. Advanced monitoring tools such as esophageal pressure (P_es_) or diaphragm electrical activity (EAdi) offer the benefit of detecting asynchrony; however, the use of these monitoring tools is limited due to invasiveness and its cost [6, 13]. Recent studies showed the feasibility of machine learning and computer algorithm to analyze P_aw_ and airflow waveforms and to identify patient ventilator asynchrony [14–16]. IntelliSync + software (Hamilton Medical AG, Bonaduz, Switzerland) is a relatively new ventilation technology that continuously analyzes P_aw_ and airflow waveforms by detecting the initiation and end of inspiration in real-time that can improve patient ventilator interaction; however, evidence of this software for improving patient ventilator synchronization is limited. The aim of this study was to evaluate the effect of IntelliSync + software on patient ventilator synchronization and breathing patterns in mechanically ventilated patients with PSV mode.

## Methods

### Study design and population

A randomized crossover physiological study was conducted in the Respiratory Intensive Care Unit, Department of Medicine, Faculty of Medicine Siriraj Hospital, Mahidol University, Bangkok, Thailand, from September 2020 to February 2022. The study was approved by the Siriraj Institutional Review Board (certificate of approval No. Si632/2020; date of approval 22/07/2020) and was registered in the Thai Clinical Trial Registry (date of registration: 28/05/2020, the registration number: TCTR20200528003). Written informed consent to participate was obtained from each subject or their relatives. This research project was supported by the Faculty of Medicine Siriraj Hospital, Mahidol University (grant number [IO]R016331065(fund3)).

Mechanically ventilated patients with age *≥* 18 years who were ventilated in the PSV mode with the following criteria: pressure support level *≤* 16 cmH_2_O, positive end-expiratory pressure (PEEP) *≤* 10 cmH_2_O, and oxygen fraction (FiO_2_) *≤* 0.6 were enrolled. Patients were excluded if they met any of the following criteria under PSV: unstable hemodynamics (systolic blood pressure > 180 mmHg or < 90 mmHg, diastolic blood pressure > 100 mmHg or < 60 mmHg, heart rate > 140 beats/minute or < 60 beats/minute, or any sign of poor tissue perfusion), respiratory rate > 35 breaths/minute, oxygen saturation by pulse oximetry (SpO_2_) < 92%, severe acid-base disturbance (arterial pH < 7.30 or > 7.55), tracheostomized patient, contraindication for esophageal balloon catheter insertion, or pregnant woman.

### Ventilator and equipment

The Hamilton S1 (Hamilton Medical AG, Bonaduz, Switzerland) with IntelliSync + software was used in this study. IntelliSync + software can be activated in the trigger phase (inspiratory trigger synchronization: I-sync), cycling phase (cycle synchronization: C-sync), or both phases (inspiratory trigger and cycle synchronization: I/C-sync). Airflow was measured with a pneumotachograph placed between the endotracheal tube and the Y-piece of the ventilator and connected to a differential pressure transducer (MP150, BIOPAC Systems, Gotela, California, USA). P_aw_ was measured between the endotracheal tube and the pneumotachograph using a pressure transducer (MP150, BIOPAC Systems, Gotela, California, USA).

An esophageal balloon catheter (CooperSurgical, Trumbull, Connecticut, USA) was inserted through the nose and positioned in the lower third of the esophagus. The balloon was filled with 1 mL of air according to the manufacturer’s instructions and connected to a pressure transducer (BIOPAC Systems, Gotela, California, USA). The position of the esophageal balloon was checked by applying gentle pressure on the abdomen to verify the absence of fluctuating gastric pressure, then an occlusion test was performed to confirm the position [17, 18]. P_es_ was recorded with an MP150 Data Acquisition System (BIOPAC Systems, Gotela, California, USA).

The analog signals for airflow, P_aw_, and P_es_ were digitized at a sampling rate of 100 Hz and stored on a laptop for subsequent offline analysis using AcqKnowledge software (BIOPAC, Systems, Gotela, California, USA).

### Study protocol

**Fig. 1:**
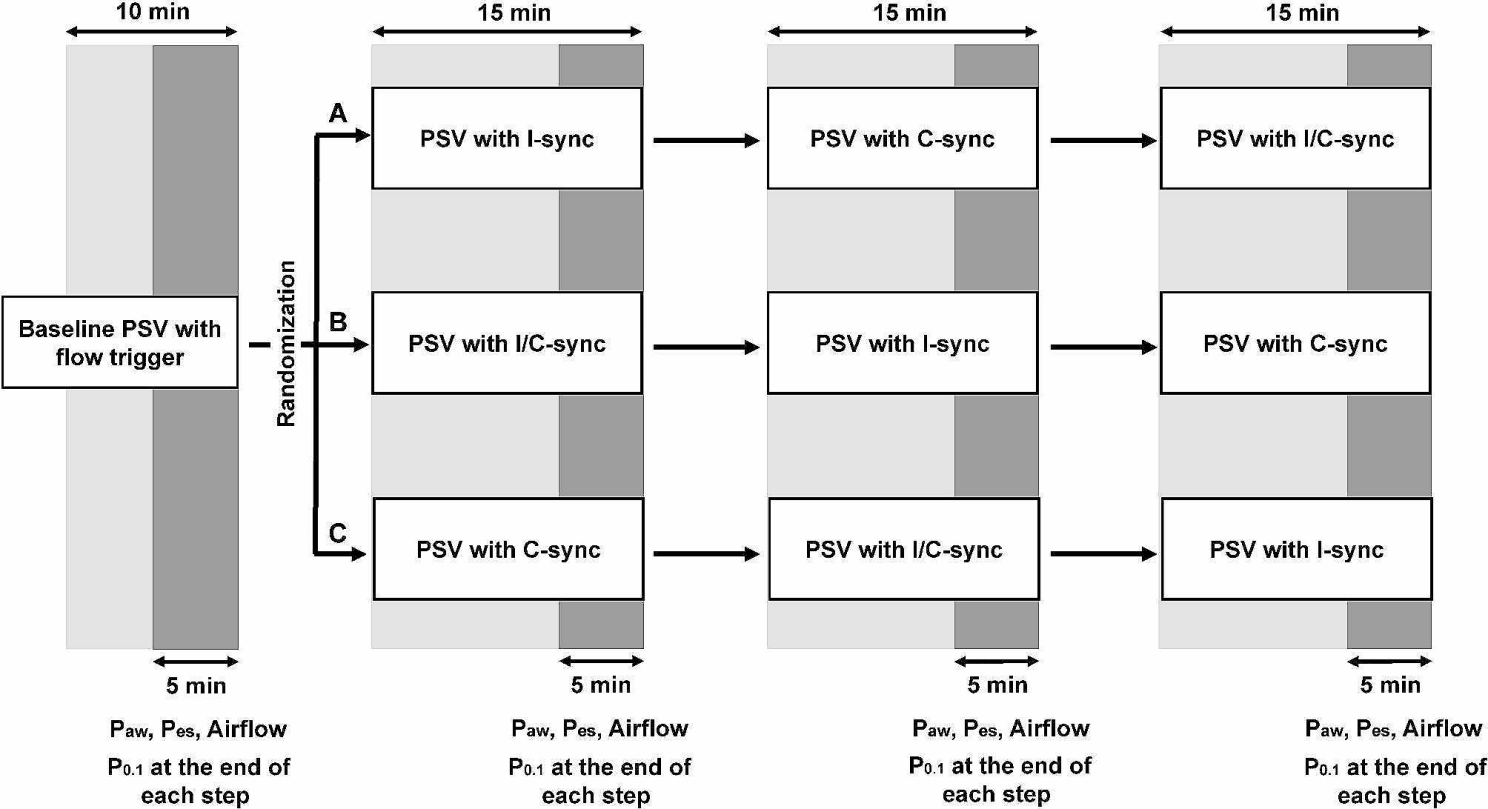
Study protocol. C-sync – cycle synchronization, I-sync – inspiratory trigger synchronization, I/C-sync – inspiratory trigger and cycle synchronization, P_aw_ – airway pressure, P_es_ – esophageal pressure, PSV – pressure support ventilation, P_0.1_ – airway occlusion pressure at 0.1 s

The patients were studied in a semi-recumbent position. At baseline, patients were ventilated with PSV mode using their clinical settings with conventional flow trigger and cycling for 10 min, then randomly assigned using a sealed opaque envelope to one of the following sequences: Sequence A – I-sync → C-sync → I/C sync, Sequence B – I/C-sync → I-sync → C-sync, or Sequence C – C-sync → I/C-sync → I-sync (Fig. 1). Each step was applied for 15 min (the first 10 min were devoted to ensure the full adaptation of the patient to the mode, and the signal acquisition was carried out during the last 5 min). Other ventilator settings were kept unchanged in all study sequences.

### Data collection

Baseline demographic and clinical data including age, sex, body mass index, and comorbidity were collected. Acute Physiologic and Chronic Health Evaluation (APACHE) II and Sequential Organ Failure (SOFA) scores were evaluated at admission. The Richmond Agitation-Sedation Scale (RASS) was assessed on the study date. During the study intervention period, blood pressure, respiratory rate, and heart rate were recorded every 5 min. SpO_2_ was continuously recorded throughout the study period. We continuously recorded P_aw_, airflow, and P_es_ waveforms for 5 min at the end of each step. The recorded waveforms were analyzed offline, with the investigator blinded to each intervention using the waveforms of the last 2 min of recording using a dedicated software program (AcqKnowledge Data Acquisition and Analysis Software, BIOPAC Systems, Gotela, California, USA). The airway occlusion pressure displayed by the ventilator at 0.1 s (P_0.1_) was also recorded for 5 consecutive breaths at the end of each intervention and the average value was reported. The ventilator automatically measured P_0.1_ breath-by-breath during pressure trigger system without airway occlusion by calculating the steepest slope of the pressure drop during an inspiratory effort and extrapolated the P_aw_ drop at 100 msec below PEEP [19].

**Fig. 2:**
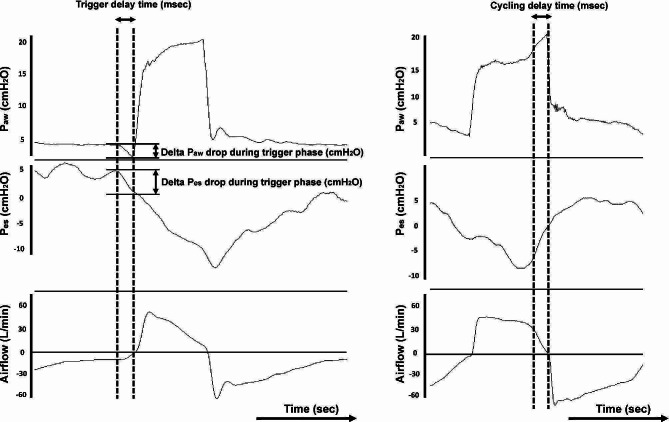
Measurement of inspiratory trigger delay time, airway pressure drop (P_aw_) and esophageal pressure drop (P_es_) during trigger phase, and cycling delay time

Inspiratory trigger delay time was defined as the time difference between the initial drop in the P_aw_ and the beginning of the ventilator delivered pressurization (Fig. 2A). Delta P_aw_ and P_es_ drops during trigger phase were calculated as the pressure difference in the initial drop in P_aw_ and P_es_ and the beginning of ventilator delivered pressurization (Fig. 2A). The cycle delay time was calculated as the time difference between the end of patient inspiration (the point of P_es_ that elapsed 25% of time from its maximum P_es_ deflection to return to baseline [20–22]) and the opening of the expiratory valve (Fig. 2B).

The asynchrony index was calculated as the number of major asynchronous breaths (ineffective effort, double triggering, and auto triggering) divided by the total number of breaths [23]. We did not take into account the premature- and delayed cycling for the asynchrony index because the cycle delay time was our objective.

### Outcomes

The primary outcome was inspiratory trigger and cycle delay time between each intervention. The secondary outcomes were delta P_aw_ drop during the trigger phase, delta P_es_ drop during trigger phase, P_0.1_, respiratory rate, SpO_2_, mean arterial pressure and heart rate between each intervention.

### Statistical analysis

The sample size was calculated based on the previous study by Mojoli and colleagues [24], IntelliSync + software significantly reduced cycle delay time compared to conventional PSV from 282 ± 315 msec to 54 ± 152 msec, using a significant level of 0.05 and a power of 80% to detect the difference between the two groups, a sample size of 14 subjects was calculated.

The Shapiro-Wilk test was used to assess the normality of the data. Continuous variables are presented as mean±standard deviation or median [interquartile range]. Categorical variables are presented as absolute numbers and percentages. For normally distributed data, we used an analysis of variance (ANOVA) with repeated measures followed by a post hoc pairwise comparison with Bonferroni correction. Nonnormally distributed data were compared using Friedman’s two-way ANOVA by ranks with a post hoc pairwise comparison. A *P* < 0.05 was considered statistically significant. Data were analyzed using PASW Statistics version 18 (SPSS, Inc., Chicago, Illinois, USA).

## Results

**Fig. 3:**
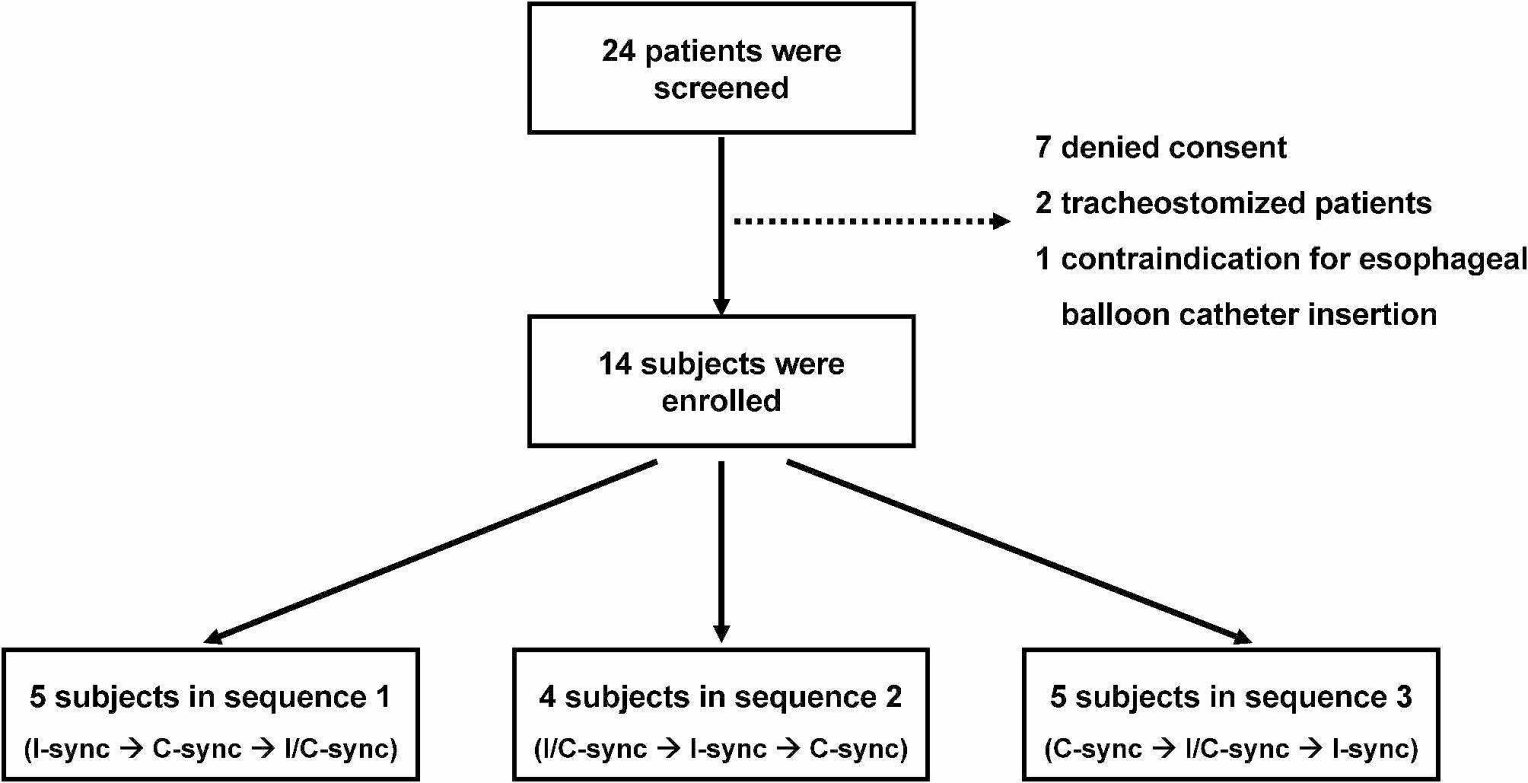
CONSORT flow diagram. C-sync – cycle synchronization, I-sync – inspiratory trigger synchronization, I/C-sync – inspiratory trigger and cycle synchronization

Fourteen mechanically ventilated patients were enrolled and analyzed (Fig. 3). The mean age was 65±15 years, and 57.1% of enrolled subjects were men. The mean scores for APACHE II and SOFA scores were 15±9 and 6±4, respectively. Pneumonia was the most common cause of acute respiratory failure in this study (71.4%). Other clinical characteristics are shown in Table 1. All patients were ventilated in PSV mode at an average pressure support level of 11±3 cmH_2_O and PEEP of 6±2 cmH_2_O. Other baseline physiological variables are shown in Table 2.

**Table 1:** Baseline demographic and clinical characteristics

Variables	*N* = 14
Age, years	65±15
Male gender, n (%)	8 (57.1)
Body mass index, kg/m^2^	27.8±8.6
Comorbidity	
• Hypertension	10 (71.4)
• Diabetes	6 (42.9)
• Cardiovascular disease	4 (28.6)
• Chronic respiratory disease	4 (28.6)
• Chronic kidney disease	4 (28.6)
APACHE II score on admission	15±9
SOFA score on admission	6±4
RASS score at enrollment date	0 [0–0]
Cause of acute respiratory failure	
• Pneumonia	10 (71.4)
• Exacerbation of COPD	1 (7.1)
• Acute pulmonary embolism	1 (7.1)
• Others	2 (14.3)
Duration of mechanical ventilation before enrollment, days	7 [3–13]

Data are presented as mean±standard deviation, median [interquartile range], or absolute number (percentage)

APACHE – Acute Physiologic and Chronic Health Evaluation, COPD – chronic obstructive pulmonary disease, RASS – Richmond Agitation and Sedation Scale, SOFA – Sequential Organ Failure Assessment

**Table 2:** Baseline ventilator settings and physiological variables

Variables	*N* = 14
Ventilator settings	
• Flow trigger, L/min	2 [2–2]
• Pressure support, cmH_2_O	11±3
• Positive end-expiratory pressure, cmH_2_O	6±2
• Flow cycling (of peak inspiratory flow), %	25 [25–25]
• FiO_2_	0.40 [0.30–0.40]
• Pressure rise time, msec	50 [50–50]
Vital signs and respiratory variables	
• Respiratory rate, breaths/minute	23±7
• Mean arterial pressure, mmHg	91±16
• Heart rate, beats/min	90±19
• Tidal volume, mL	389±71
• Minute ventilation, L/min	9.3±2.4
Gas exchange	
• pH	7.41±0.08
• PaCO_2_, mmHg	37.7 [34.7–43.5]
• PaO_2_, mmHg	99.4±29.3
• SpO_2_, %	99 [97–100]

Data are presented as mean±standard deviation or median [interquartile range]

FiO_2_ – oxygen fraction, PaCO_2_ – arterial partial pressure of carbon dioxide, PaO_2_ – arterial partial pressure of oxygen, SpO_2_ – oxygen saturation by pulse oximetry

**Fig. 4:**
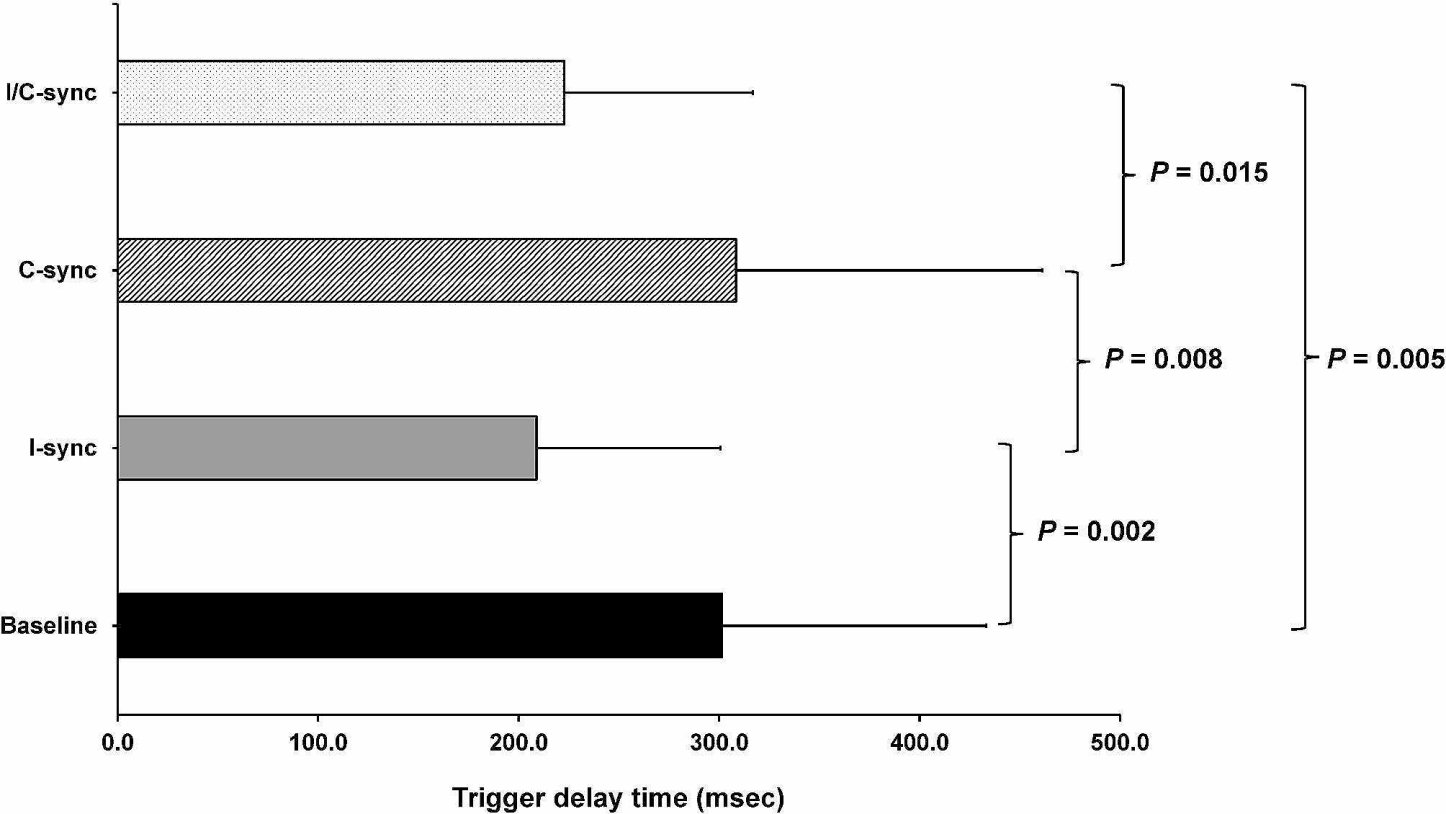
Inspiratory trigger delay time between each intervention. C-sync – cycle synchronization, I-sync – inspiratory trigger synchronization, I/C-sync – inspiratory trigger and cycle synchronization

The inspiratory trigger delay time was significantly shorter with I-sync and I/C-sync compared to baseline (208.9±91.7 vs. 301.4±131.7 msec; *P* = 0.002 and 222.8±94.0 vs. 301.4±131.7 msec; *P* = 0.005; respectively) (Fig. 4). The I-sync and I/C-sync groups had significantly shorter trigger delay time compared to C-sync group (208.9±91.7 vs. 308.4±153.0 msec; *P* = 0.008 and 222.8±94.0 vs. 308.4±153.0 msec; *P* = 0.015; respectively). Change in trigger delay time from baseline to each mode is shown in Supplementary Fig. 1 (Additional File 1).

There was a trend towards shorter cycle delay time between C-sync and I/C-sync compared to baseline; however, no statistically significant differences were observed (Table 3). Change in cycle delay time from baseline to each mode is shown in Supplementary Fig. 2 (Additional File 1). No significant differences in the modified asynchrony index, P_0.1_ and other physiological variables were found between the groups (Table 3).

**Table 3:** Waveform analysis and physiological variables between each intervention

Variables	Baseline	I-sync	C-sync	I/C-sync	*P*-value
Trigger delay time, msec	301.4±131.7	208.9±91.7***,****	308.4±153.0	222.8±94.0^#,##^	0.024
Delta P_aw_ drop during trigger phase, cmH_2_O	-0.9±0.5	-0.7±0.5	-1.2±0.8	-0.7±0.4*	0.003
Delta P_es_ drop during the trigger phase, cmH_2_O	-2.1±1.1	-2.0±2.7^###^	-2.8±3.2	-1.8±2.2**	0.021
Cycle delay time, msec	265.4±194.8	231.6±186.4	158.1±237.8	191.4±174.4	0.509
P_0.1_, cmH_2_O	1.6±0.8	1.5±0.6	1.6±0.7	1.7±0.7	0.752
Respiratory rate, breaths/min	23±6	21±6	22±6	25±7	0.285
Mean arterial pressure, mmHg	92±15	90±17	90±13	91±14	0.830
Heart rate, beats/min	88±17	87±19	88±17	88±17	0.980
SpO_2_, %	100 [97–100]	99 [98–100]	100 [98–100]	99 [97–100]	0.275
Modified asynchrony index, %	0.0 [0.0-1.3]	0.0 [0.0-0.5]	0.0 [0.0–0.0]	0.0 [0.0-5.3]	0.923

Data are presented as mean±standard deviation or median [interquartile range]

C-sync – cycle synchronization, I-sync – inspiratory trigger synchronization, I/C-sync – inspiratory trigger and cycle synchronization, P_aw_ – airway pressure, P_es_ – esophageal pressure, P_0.1_ – airway occlusion pressure at 0.1 s, SpO_2_ – oxygen saturation by pulse oximetry

*I/C-sync vs. C-sync (P-value = 0.028), ** I/C-sync vs. C-sync (P-value = 0.011), ***I-sync vs. baseline (P-value = 0.002), ****I-sync vs. C-sync (P-value = 0.008), ^#^I/C-sync vs. C-sync (P-value = 0.015), ^##^I/C-sync vs. baseline (P-value = 0.005), ^###^I-sync vs. C-sync (P-value = 0.027)

There was no significant difference in delta P_aw_ drop and P_es_ drop during trigger phase between the baseline and the other three intervention groups; however, the I/C-sync group had significantly lower delta P_aw_ and P_es_ drop during trigger phase compared to C-sync group (-0.7±0.4 vs. -1.2±0.8 cmH_2_O; *P* = 0.028 and − 1.8±2.2 vs. -2.8±3.2 cmH_2_O; *P* = 0.011, respectively). Changes in delta P_aw_ and P_es_ drop from baseline to each mode are shown in Supplementary Figs. 3 and 4 (Additional File 1).

No adverse event was observed during the study period and all subjects tolerated both interventions until the end of the study.

## Discussion

Our study demonstrated that IntelliSync + software significantly improved inspiratory trigger delay time compared to baseline ventilator settings. In addition, the drop in P_aw_ and P_es_ during trigger phase was significantly better with I/C-sync compared to C-sync in mechanically ventilated patients receiving PSV mode. However, there was no significant difference in cycle delay time between IntelliSync + software and the conventional flow cycling system.

Patient ventilator asynchrony commonly occurs in mechanically ventilated patients, especially during PSV mode and it was associated with poor clinical outcomes [7–9]. Recently, many dedicated machine-learning software designed to continuously detect patient ventilator asynchrony have been developed. A systematic review demonstrated that these algorithms or software had high sensitivity and specificity to detect patient ventilator asynchrony compared to the reference standard using P_es_ or EAdi [25]. IntelliSync + software (Hamilton Medical AG, Bonaduz, Switzerland) is a new technology to detect the initiation and the end of inspiration by analyzing P_aw_ and airflow waveforms. This waveform method has been evaluated in 16 mechanically ventilated patients and showed a precise assessment of the timing of patient spontaneous activity during PSV mode [23]. In addition, it was highly reproducible and reliable to detect both major and minor asynchronies. IntelliSync + software can be operated during the inspiratory and/or expiratory phases that can improve patient ventilator interaction; however, the data regarding the accuracy and feasibility of this software are scant. A study by Mojoli and colleagues in 15 mechanically ventilated patients with PSV mode demonstrated that IntelliSync + software significantly reduced cycling delay time and ineffective efforts compared to baseline PSV support [26]. In addition, increasing pressure support worsened patient ventilator interaction, but IntelliSync + software was superior to the setting by the expert in terms of patient ventilator synchronization.

Our findings confirm that IntelliSync + software is beneficial in improving patient ventilator interaction by reducing the inspiratory trigger delay time with I-sync and I/C-sync compared to conventional PSV. In our study, we did not evaluate the effect of IntelliSync + software on inspiratory effort so the reduction of inspiratory trigger delay time with IntelliSync + software approximately 100 msec might not have the impact on clinical outcome. However, other clinical studies using a proportional mode of ventilation such as neurally adjust ventilatory assist or proportional assist ventilation demonstrated that these modes significantly improved inspiratory trigger delay time (varying from 100 to 150 msec) and reduced asynchrony index and inspiratory effort measured by pressure-time product compared to PSV [27–31]. In addition, IntelliSync + software reduced the drop in P_aw_ and P_es_ during the trigger phase that may help alleviate the trigger work of breathing but the impact on clinical outcomes was beyond the scope of our study and it should be evaluated in the future. However, an improvement in cycle delay time was not observed in the present study, although there was a trend toward a shorter cycle delay time with C-sync. The setting of conventional flow cycling in our study was quite short at the baseline, which may explain why the reduction in cycle delay time was not observed. In addition, the small sample size in the present study may not be enough to detect the difference in the cycle delay time between C-sync and other interventions. Larger studies are needed to evaluate the effect of IntelliSync + software in terms of patient ventilator interaction and clinical outcomes.

### Limitations

Our study has some limitations. First, this study had a small number of enrolled subjects. Second, the time spent on each intervention was relatively short. Third, our study was performed in the PSV mode, so our findings might not be generalizable to other modes of ventilation. Finally, this study was designed to evaluate the physiological effects of IntelliSync + software, but not on clinical outcomes. Future studies are needed to evaluate the longer effect of IntelliSync + software on patient-ventilator interaction and its impact on clinical outcomes.

## Conclusions

IntelliSync + software improved inspiratory trigger delay time compared to conventional flow trigger system during PSV mode. However, no significant improvement in cycle delay time and other physiological variables was observed with IntelliSync + software.

### Electronic supplementary material

Below is the link to the electronic supplementary material.

Supplementary Material 1

## Data Availability

The datasets used and/or analyzed during the current study are available from the corresponding author on reasonable request.

## Abbreviations

C-sync: Cycle synchronization
FiO_2_: Oxygen fraction
ICU: Intensive care unit
I-sync: Inspiratory trigger synchronization
I/C-sync: Inspiratory trigger and cycle synchronization
PaCO_2_: Arterial partial pressure of carbon dioxide
PaO_2_: Arterial partial pressure of oxygen
P_aw_: Airway pressure
PEEP: Positive end-expiratory pressure
P_es_: Esophageal pressure
PSV: Pressure support ventilation
P_0.1_: Airway occlusion pressure at 0.1 s
SpO_2_: Oxygen saturation by pulse oximetry

## References

[1] Esteban A, Anzueto A, Frutos F, Alía I, Brochard L, Stewart TE (2002). Characteristics and outcomes in adult patients receiving mechanical ventilation: a 28-day international study. JAMA.

[2] Esteban A, Frutos-Vivar F, Muriel A, Ferguson ND, Peñuelas O, Abraira V (2013). Evolution of mortality over time in patients receiving mechanical ventilation. Am J Respir Crit Care Med.

[3] van Haren F, Pham T, Brochard L, Bellani G, Laffey J, Dres M (2019). Spontaneous breathing in early acute respiratory distress syndrome: insights from the large observational study to UNderstand the global impact of severe Acute Respiratory FailurE study. Crit Care Med.

[4] Rittayamai N, Katsios CM, Beloncle F, Friedrich JO, Mancebo J, Brochard L (2015). Pressure-controlled vs volume-controlled ventilation in Acute Respiratory failure: a physiology-based narrative and systematic review. Chest.

[5] Hess DR (2005). Ventilator waveforms and the physiology of pressure support ventilation. Respir Care.

[6] Dres M, Rittayamai N, Brochard L (2016). Monitoring patient-ventilator asynchrony. Curr Opin Crit Care.

[7] de Wit M, Miller KB, Green DA, Ostman HE, Gennings C, Epstein SK (2009). Ineffective triggering predicts increased duration of mechanical ventilation. Crit Care Med.

[8] Blanch L, Villagra A, Sales B, Montanya J, Lucangelo U, Luján M (2015). Asynchronies during mechanical ventilation are associated with mortality. Intensive Care Med.

[9] Thille AW, Rodriguez P, Cabello B, Lellouche F, Brochard L (2006). Patient-ventilator asynchrony during assisted mechanical ventilation. Intensive Care Med.

[10] Vaporidi K, Babalis D, Chytas A, Lilitsis E, Kondili E, Amargianitakis V (2017). Clusters of ineffective efforts during mechanical ventilation: impact on outcome. Intensive Care Med.

[11] Pham T, Telias I, Piraino T, Yoshida T, Brochard LJ (2018). Asynchrony consequences and Management. Crit Care Clin.

[12] Colombo D, Cammarota G, Alemani M, Carenzo L, Barra FL, Vaschetto R (2011). Efficacy of ventilator waveforms observation in detecting patient-ventilator asynchrony. Crit Care Med.

[13] Mauri T, Yoshida T, Bellani G, Goligher EC, Carteaux G, Rittayamai N (2016). Esophageal and transpulmonary pressure in the clinical setting: meaning, usefulness and perspectives. Intensive Care Med.

[14] Gholami B, Phan TS, Haddad WM, Cason A, Mullis J, Price L (2018). Replicating human expertise of mechanical ventilation waveform analysis in detecting patient-ventilator cycling asynchrony using machine learning. Comput Biol Med.

[15] Chen C-W, Lin W-C, Hsu C-H, Cheng K-S, Lo C-S (2008). Detecting ineffective triggering in the expiratory phase in mechanically ventilated patients based on airway flow and pressure deflection: feasibility of using a computer algorithm. Crit Care Med.

[16] Hoff FC, Tucci MR, Amato MBP, Santos LJ, Victorino JA (2014). Cycling-off modes during pressure support ventilation: effects on breathing pattern, patient effort, and comfort. J Crit Care.

[17] Akoumianaki E, Maggiore SM, Valenza F, Bellani G, Jubran A, Loring SH (2014). The application of esophageal pressure measurement in patients with respiratory failure. Am J Respir Crit Care Med.

[18] Jonkman AH, Telias I, Spinelli E, Akoumianaki E, Piquilloud L (2023). The oesophageal balloon for respiratory monitoring in ventilated patients: updated clinical review and practical aspects. Eur Respir Rev.

[19] Brenner M, Mukai DS, Russell JE, Spiritus EM, Wilson AF (1990). A new method for measurement of airway occlusion pressure. Chest.

[20] Rittayamai N, Beloncle F, Goligher EC, Chen L, Mancebo J, Richard J-CM (2017). Effect of inspiratory synchronization during pressure-controlled ventilation on lung distension and inspiratory effort. Ann Intensive Care.

[21] Grieco DL, Menga LS, Raggi V, Bongiovanni F, Anzellotti GM, Tanzarella ES (2020). Physiological comparison of High-Flow Nasal Cannula and Helmet Noninvasive Ventilation in Acute Hypoxemic Respiratory failure. Am J Respir Crit Care Med.

[22] Rittayamai N, Phuangchoei P, Tscheikuna J, Praphruetkit N, Brochard L (2019). Effects of high-flow nasal cannula and non-invasive ventilation on inspiratory effort in hypercapnic patients with chronic obstructive pulmonary disease: a preliminary study. Ann Intensive Care.

[23] Mojoli F, Pozzi M, Orlando A, Bianchi IM, Arisi E, Iotti GA (2022). Timing of inspiratory muscle activity detected from airway pressure and flow during pressure support ventilation: the waveform method. Crit Care.

[24] Velasquez T, Mackey G, Lusk J, Kyle UG, Fontenot T, Marshall P (2016). ESICM LIVES 2016: part three. Intensive Care Med Experimental.

[25] Bandeira M, Almeida A, Melo L, de Moura PH, Ribeiro Silva EO, Silva J (2021). Accuracy of algorithms and Visual Inspection for Detection of Trigger Asynchrony in critical patients: a systematic review. Crit Care Res Pract.

[26] Mojoli F, Orlando A, Bianchi IM, Puce R, Arisi E, Salve G (2022). Waveforms-guided cycling-off during pressure support ventilation improves both inspiratory and expiratory patient-ventilator synchronisation. Anaesth Crit Care Pain Med.

[27] Piquilloud L, Vignaux L, Bialais E, Roeseler J, Sottiaux T, Laterre P-F (2011). Neurally adjusted ventilatory assist improves patient-ventilator interaction. Intensive Care Med.

[28] Piquilloud L, Tassaux D, Bialais E, Lambermont B, Sottiaux T, Roeseler J (2012). Neurally adjusted ventilatory assist (NAVA) improves patient-ventilator interaction during non-invasive ventilation delivered by face mask. Intensive Care Med.

[29] Spahija J, de Marchie M, Albert M, Bellemare P, Delisle S, Beck J (2010). Patient-ventilator interaction during pressure support ventilation and neurally adjusted ventilatory assist. Crit Care Med.

[30] Vasconcelos R, dos Melo S, de Sales LH, Marinho RP, Deulefeu LS, Reis FC (2013). Effect of an automatic triggering and cycling system on comfort and patient-ventilator synchrony during pressure support ventilation. Respiration.

[31] Costa R, Spinazzola G, Cipriani F, Ferrone G, Festa O, Arcangeli A (2011). A physiologic comparison of proportional assist ventilation with load-adjustable gain factors (PAV+) versus pressure support ventilation (PSV). Intensive Care Med.

