# Supplementary material for: The effects of real-time waveform analysis software on patient ventilator synchronization during pressure support ventilation: a randomized crossover physiological study
Source: BMC Pulm Med. 2024 May 1;24:212. doi: 10.1186/s12890-024-03039-0 (PMC11064376; doi:10.1186/s12890-024-03039-0)

**Supplementary Fig. 1** Change of trigger delay time from baseline to A) I-sync, B) C-sync, and C) I/C sync. C-sync – cycle synchronization, I-sync – inspiratory synchronization, I/C-sync – inspiratory and cycle synchronization.


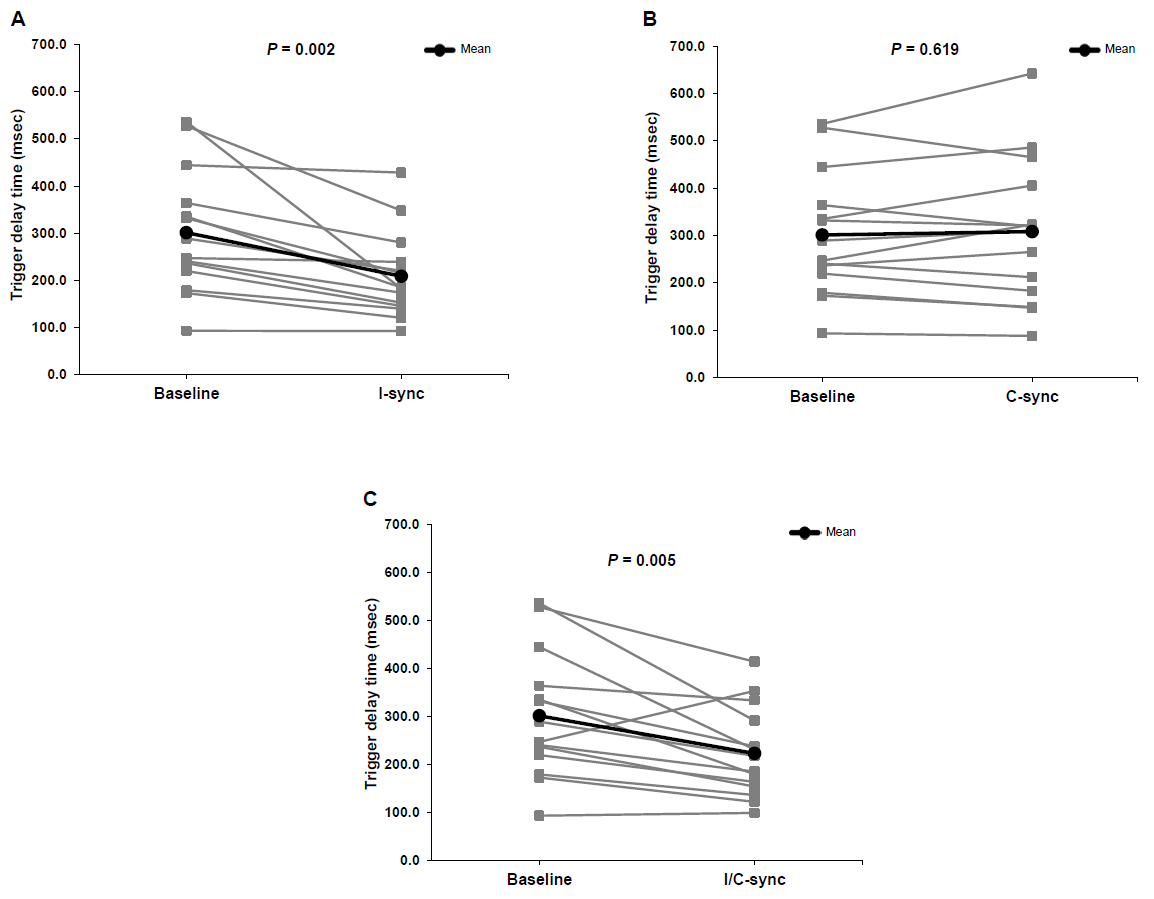


**Supplementary Fig. 2** Change of cycle delay time to A) I-sync, B) C-sync, and C) I/C sync. C-sync – cycle synchronization, I-sync – inspiratory synchronization, I/C-sync – inspiratory and cycle synchronization.


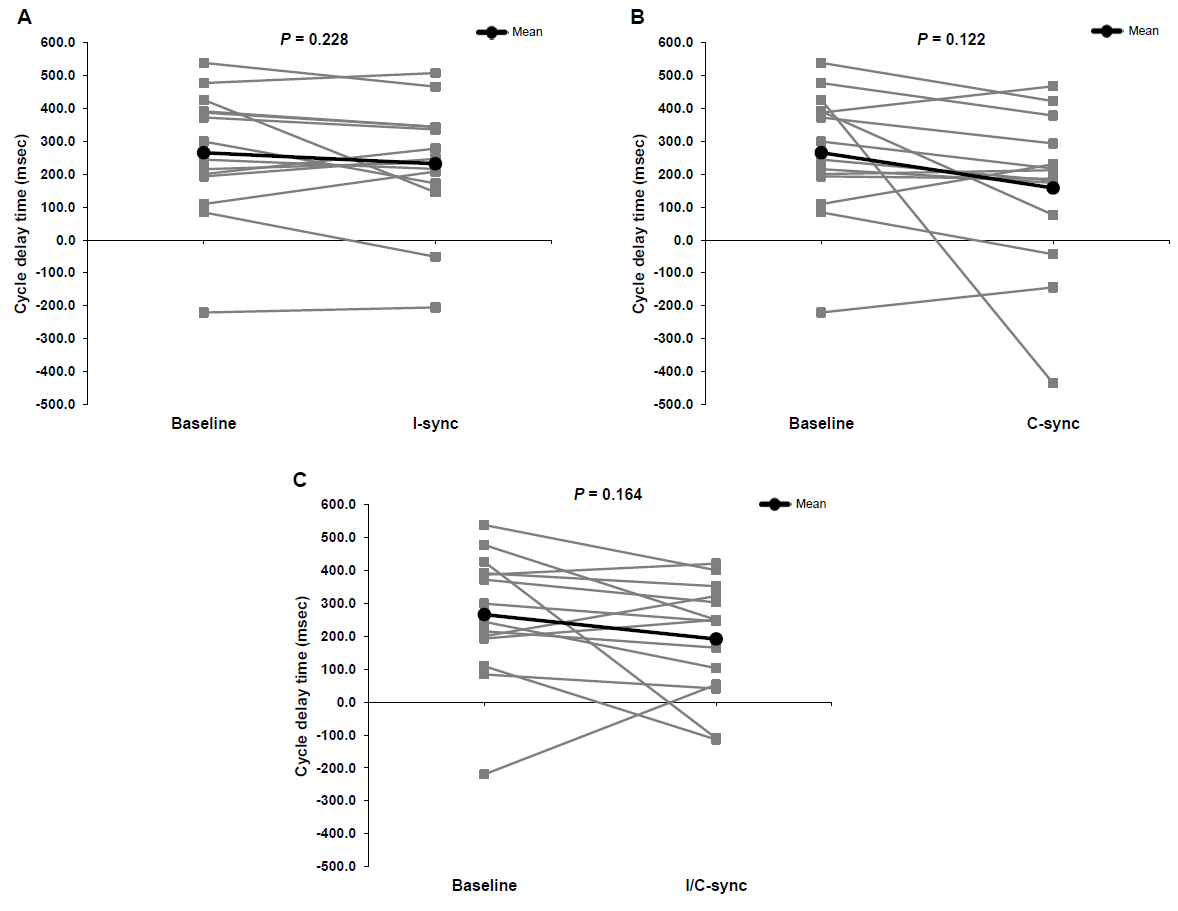


**Supplementary Fig. 3** Change of delta Paw drop from baseline to A) I-sync, B) C-sync, and C) I/C sync. C-sync – cycle synchronization, I-sync – inspiratory synchronization, I/C-sync – inspiratory and cycle synchronization.


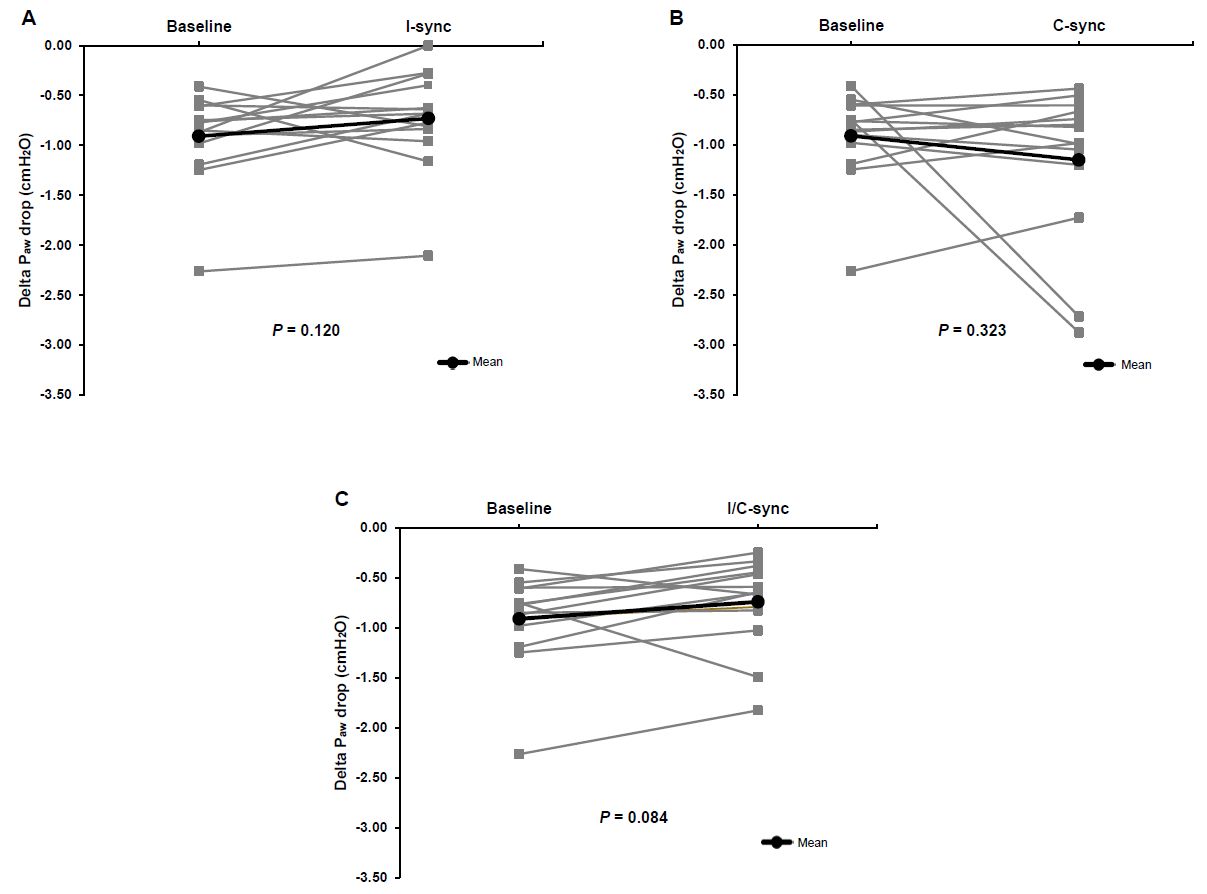


**Supplementary Fig. 4** Change of delta Pes drop from baseline to to A) I-sync, B) C-sync, and C) I/C sync. C-sync – cycle synchronization, I-sync – inspiratory synchronization, I/C-sync – inspiratory and cycle synchronization.


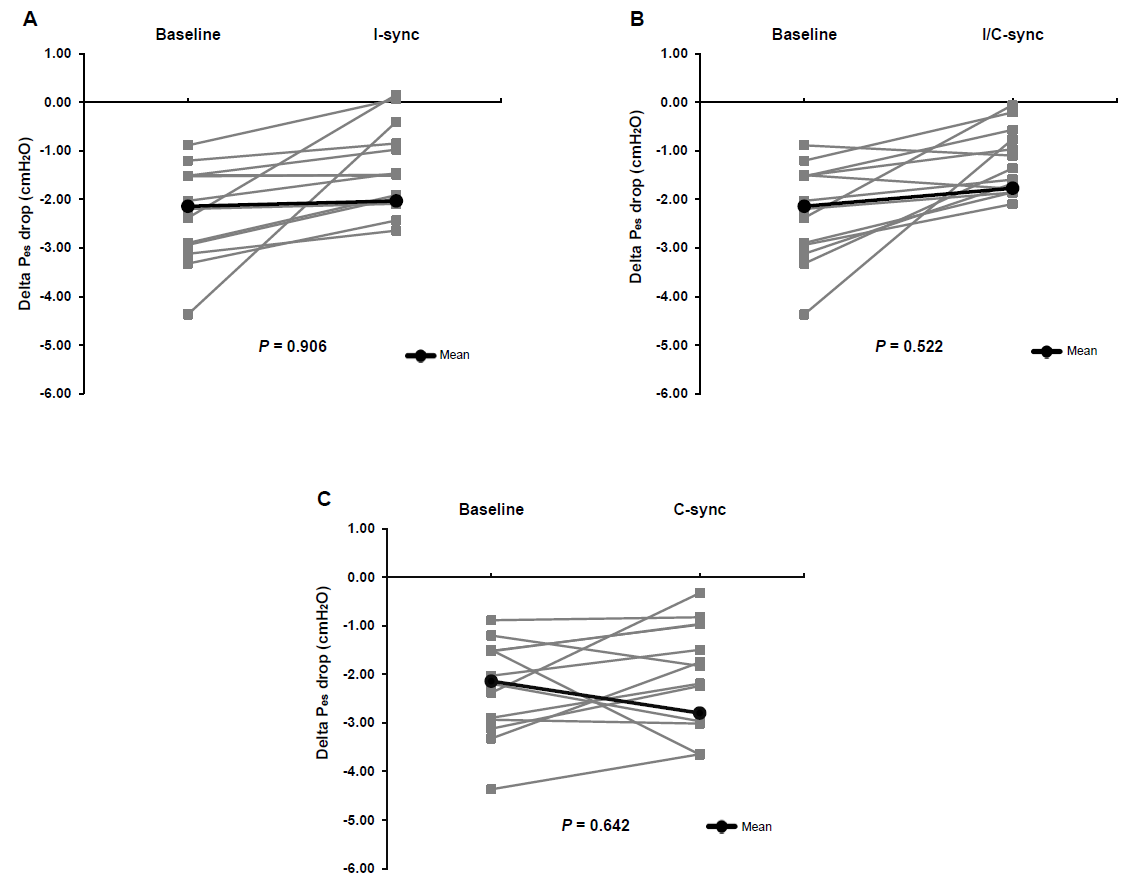

Supplement: Supplementary file 1 — Supplementary Material 1 [file 12890_2024_3039_MOESM1_ESM.docx]
